# Spatial and Temporal Oxygen Dynamics in Macrofaunal Burrows in Sediments: A Review of Analytical Tools and Observational Evidence

**DOI:** 10.1264/jsme2.ME12182

**Published:** 2013-04-16

**Authors:** Hisashi Satoh, Satoshi Okabe

**Affiliations:** 1 Division of Environmental Engineering, Faculty of Engineering, Hokkaido University, North-13, West-8, Sapporo 060–8628, Japan

**Keywords:** bioturbated sediment, macrofaunal burrow, O_2_ dynamics, O_2_ microsensor, O_2_ planar optode

## Abstract

The availability of benthic O_2_ plays a crucial role in benthic microbial communities and regulates many important biogeochemical processes. Burrowing activities of macrobenthos in the sediment significantly affect O_2_ distribution and its spatial and temporal dynamics in burrows, followed by alterations of sediment microbiology. Consequently, numerous research groups have investigated O_2_ dynamics in macrofaunal burrows. The introduction of powerful tools, such as microsensors and planar optodes, to sediment analysis has greatly enhanced our ability to measure O_2_ dynamics in burrows at high spatial and temporal resolution with minimal disturbance of the physical structure of the sediment. In this review, we summarize recent studies of O_2_-concentration measurements in burrows with O_2_ microsensors and O_2_ planar optodes. This manuscript mainly focuses on the fundamentals of O_2_ microsensors and O_2_ planar optodes, and their application in the direct measurement of the spatial and temporal dynamics of O_2_ concentrations in burrows, which have not previously been reviewed, and will be a useful supplement to recent literature reviews on O_2_ dynamics in macrofaunal burrows.

## Introduction

In aquatic ecosystems, it is obvious that the volume of the water column far exceeds the volume of the upper-surface layer of benthic sediment. However, ecological processes in an aquatic system, such as the biogeochemical carbon, nitrogen, phosphorus, and sulfur cycles, are predominantly regulated by microbial activities in the surface layer of the sediment, typically with depths of a few millimeters or centimeters, rather than by the integrated microbial activity of the entire water column (2, 3, 4, 24, 70, 77, 78, 83, 84, 104, 144, 145). This is because volume-specific microbial activity in the sediment surface, which hosts a high density of microorganisms, is typically 100 to 1,000 times higher than that in the water column (37). For example, it was estimated that up to 80% of the nitrogen needed by primary producers in shallow seas was provided by remineralization processes in the sediment (19).

Because an estuary is a transition zone between a river and an ocean, it is subject not only to marine influences (*e.g.*, tides, waves, and influx of nutrients and saline water) but also riverine influences (*e.g.*, fresh-water flow, influx of particles, and natural and anthropogenic input of nutrients, metals, and organic compounds) (60, 127, 143). Inflow of both seawater and freshwater provides high levels of organic matter and nutrients in the sediment surface of an estuary. Moreover, the reduction in water flow in an estuary accelerates the accumulation of organic matter and nutrients on the sediment surface. In particular, this makes estuaries the most productive natural habitats among aquatic ecosystems (54, 102, 103). In addition, estuaries are often heavily polluted by biodegradable organic matter, nutrients, heavy metals, PCBs, hydrocarbons, pesticides and other micro-organic contaminants (47, 61, 66, 69, 93, 98, 140, 144). As a result, estuarine sediments can act as a source or sink for environmentally relevant organic compounds, gases (O_2_, CO_2_, CH_4_, and N_2_O) and inorganic compounds (NH_4_^+^, NO_2_^−^, NO_3_^−^, PO_43_^−^, SO_42_^−^, and H_2_S) in the aquatic ecosystem (21, 61, 77, 78). These gases and ions have a profound impact on water and air quality, and life in the biosphere (2, 5, 8, 9). Thus, it is highly important to quantify benthic turnover rates of organic and inorganic compounds, and to investigate the parameters regulating these processes.

It has long been recognized that by redistributing particles and modifying water fluxes via bioturbation and bioirrigation, macrofaunas living in the sediment (*i.e.*, macrobenthos) have a significant effect on the physical, chemical and biological properties of the substratum and the interstitial water in these sediments. There have been many papers describing the impact of macrobenthos on elemental (C and N) cycles and on the enhanced transport and turnover reactions of organic and inorganic compounds and trace contaminants in sediments (7, 33, 37, 52, 56, 57, 61, 63, 65, 70, 74, 80, 122, 133). In contrast, surprisingly little attention, particularly in review papers, has been paid to the description of O_2_ concentrations and dynamics in macrofaunal burrows.

O_2_ is a key molecule for biogeochemical and metabolic processes occurring in the sediment (30, 37). It is produced by oxygenic photosynthetic organisms (cyanobacteria, algae and plants) in the presence of light, and is consumed as the preferred terminal electron acceptor in the biological breakdown of organic and inorganic compounds, as it is the most energetically favorable electron acceptor for facultative aerobic microorganisms (99). Therefore, a lack of available O_2_ can have a serious impact on aerobic microorganisms and macrobenthos. On the other hand, higher O_2_ levels are critical for anaerobic microorganisms and processes, as well as for aerobic organisms, as the formation of reactive oxygen species can interfere with cellular processes. The spatial distribution of O_2_ in the sediment can thus strongly affect biogeochemical processes and microbial community structures.

O_2_ rarely penetrates deeper parts of the sediment due to the presence of a diffusive boundary layer at the sediment–water interface, the high microbial- and chemical-consumption rates of dense bacterial communities, and diffusion resistance in the sediment, as well as low solubility in water (77, 78, 105). Typically, the O_2_ penetration depth is approximately a few millimeters and the boundary between the oxic and anoxic layers is usually found as a well-defined interface in the sediment surface (78). O_2_ concentration can vary strongly and dynamically depending on the activity of the macrobenthos and in response to environmental parameters such as temperature, light, liquid flow, and the availability of organic compounds and nutrients at the micrometer to millimeter scale (6, 30, 37, 57, 64, 83, 88, 90, 105, 123, 124). Precise quantification of O_2_ distribution and dynamics is a prerequisite for understanding the regulation of biogeochemical cycles in sediments. Such spatial and temporal O_2_ dynamics in sediments has been successfully assessed with O_2_ microsensors and O_2_ planar optodes (126).

This review aims to summarize the current knowledge on spatial and temporal O_2_ dynamics in macrofaunal burrows in sediments. Effects of bioturbation on solute transport, organic-matter mineralization activities, and biogeochemical cycles have been studied extensively (33, 37, 56, 57, 61, 70, 74, 132, 133, 141), and it is beyond the scope of this review paper to cover the entire literature on this subject. Our goals are: (i) to present the principles behind and applications of O_2_ microsensors and O_2_ planar optodes as tools for O_2_ concentration measurements in macrofaunal burrows in bioturbated sediments at high spatial (sub-mm scale) and temporal (a few seconds) resolution; (ii) to provide experimental examples of direct measurements of spatial and temporal O_2_ dynamics in such burrows; and (iii) to summarize the current studies of O_2_ concentration measurements in burrows using O_2_ microsensors and O_2_ planar optodes.

## Effects of bioturbation on sediment characteristics

Bioturbation is the activity of macrobenthos living in sediments to disperse sediment particles by relocation, tube construction, burrowing, and feeding. Macrobenthos living in tubes and burrows frequently or intermittently irrigate their burrows to introduce fresh oxygenated water for respiration and for suspended food particles, as well as to remove toxic metabolites; this irrigation generates liquid flow (37, 56, 76, 105, 132). Increased liquid flow in the burrow enhances solute exchange between the sediment and the overlying water column. The advective transport of solutes is more rapid than by molecular diffusion because macrobenthos increase solute flux into or out of the sediment by as much as several orders of magnitude (92).

Bioirrigation has many consequences for the physical, chemical and biological properties of the sediment. Burrowing macrobenthos alter the sediment texture and structure (*e.g.*, sediment porosity, permeability and particle size) by transporting particles (*e.g.*, suspended soil, silt, organic particles, metal oxides and bacteria) into the sediment (1, 15, 16, 70). Labile particles are used for the growth and respiration of macrobenthos and microorganisms, whereas inert particles are deposited onto the burrow wall. Macrobenthos can vary the components and concentrations of organic compounds by breaking them down. These metabolites are then used by the microorganisms living on the burrow wall.

Dissolved compounds, such as O_2_, oxidized inorganic compounds (*e.g.*, NO_2_^−^, NO_3_^−^, SO_42_^−^ and oxidized metal ions), organic compounds and nutrients, are also transported through the burrows into the deep sediment by bioirrigation (6, 15, 59, 75, 147). Their enhanced transport into the sediment allows extension of the oxic–anoxic interface into otherwise reduced sediment (43, 105). Metabolites from the macrobenthos, and reduced compounds (*e.g.*, NH_4_^+^, H_2_S and reduced iron and manganese) in the deep sediment, are removed from the burrow and into the water column in the opposite direction. Consequently, the activities of macrobenthos also affect water quality in the overlying water column.

Other effects of macrobenthos are the development of biofilms and mucus deposition on the burrow wall, which can create a highly reactive layer and alter the radial diffusion of solutes across the sediment–water interface (1). Thus, the physicochemical properties of the burrow wall are different from those on both the sediment surface and in the surrounding sediment.

The input of various types of organic compounds and excess nutrients to the burrow changes the redox conditions, generating a variety of niches for both aerobic and anaerobic microorganisms and supporting the proliferation of a large number of highly diversified microorganisms, thus enhancing their activities on the burrow wall in the deeper layers of the sediment (10, 37, 61, 62, 105). In addition, irrigation during the burrow-maintenance activities of the macrobenthos allows the transport of aerobic microorganisms from the overlying oxygenated water into burrows in deeper layers of sediment. In this way, the activities of macrobenthos can affect the abundance and activities of microorganisms on the burrow wall by providing substrates and/or by grazing and predation. Thus, bioirrigation has significant effects not only on the components and concentrations of organic and inorganic compounds, but also on the microorganisms and the rate of associated biogeochemical processes in the sediment. For instance, recent studies have shown that bacterial abundance and activity on the burrow wall were 10-fold those in the surrounding bulk sediments (61), and that the sedimentary phosphorus cycle was also strongly enhanced through bioturbation (98, 139).

More importantly, because macrobenthos activities vary temporally and spatially, bioirrigation increases heterogeneity in the sediment’s biogeochemical conditions. Bottom-dwelling macrobenthos (*e.g.*, polychaetes and insect larvae) create unique physical structures (such as burrows and tubes) in the sediment by reworking and burrowing activities. Photographs of burrows in bioturbated sediments show burrow openings, U-shaped burrows and series of tunnels and chambers (12, 42, 43, 79). Through the activity of macrobenthos, one-dimensional diagenetic stratification of physicochemical and biological microenvironments in the sediment is transformed into three-dimensional, complex and time-dependent stratification. Their burrowing activities considerably increase the area of the sediment–water interface available for diffusive solute exchange, as well as the area of oxic–anoxic boundaries in the entire sediment. Satoh *et al.* (105) estimated the specific surface area of the burrow walls in the upper 350 mm of the sediment to be 26 m^2^ m^−3^. The tubes and burrows differ in size, appearance and composition, depending on the identity, mode of life and habits, density, and depth distribution of the macrobenthos that inhabit them. The presence of thalassinidean shrimp burrows has been shown to increase the sediment surface area by up to nine times (43). Teal *et al.* (130) calculated the global volume of bioturbated sediment to be 20,700 km^3^, based on a conservative estimate of 360 million km^2^ for the ocean area. It is clear that macrobenthos provide a variety of microorganisms with a substantial number of niches in the sediment.

Because measurement of O_2_ concentrations inside the burrows is methodologically difficult, several different techniques have been tried. In early studies, O_2_ concentrations, as well as other nutrient concentrations (*e.g.*, inorganic nitrogenous compounds), in the burrows were measured using *ex situ* approaches. Koike and Mukai (53) measured concentrations of O_2_ and inorganic nitrogenous compounds in burrows occupied by the shrimps *Callianassa japonica* and *Upogebia major* by directly collecting liquid samples from inside the burrows. These studies provided only net results, integrating the spatial and temporal O_2_ dynamics of the macrofaunal activities, because the O_2_ concentration measured was the mean value throughout the burrow. Kristensen (58) monitored the O_2_ consumption of *Nereis virens*, *N. succinea* and *N. diversicolor* by monitoring incurrent and excurrent water. This method tends to underestimate the effects of macrobenthos activities because the macrobenthos under consideration were confined to an artificial tube, causing shifts in their behavior due to the stress associated with the unnatural environmental conditions. Thus, such approaches are not suitable for studies of O_2_ concentrations in real macrofaunal burrows (14). To overcome these obstacles, an electrochemical O_2_ microsensor has been employed to determine *in situ* O_2_ concentrations in and around a single macrofaunal burrow at high spatial and temporal resolution (10, 105, 121).

## Oxygen microsensors

### Characteristics

An O_2_ microsensor is a needle-shaped electrochemical sensor with a tip diameter of approximately 10 μm. Its unique characteristics allow for O_2_ concentration measurements in the sediment at very high temporal and spatial resolution. The 90% response time for a typical O_2_ microsensor is less than 1 s (85, 86, 87, 95, 96, 109). O_2_ concentration measurements in microbial communities are recommended at intervals of twice the tip diameter (112), and hence the spatial resolution of the O_2_ microsensor will be approximately 20 μm. Therefore, the O_2_ microsensor is, at present, one of the best tools for the direct *in situ* measurement of O_2_ concentrations at the sediment surface.

Three types of electrochemical microsensors are most frequently used in environmental applications: amperometric microsensors, potentiometric microsensors and micro-biosensors, which are actually amperometric microsensors that incorporate a biological or enzymatic reaction into the sensor tip (17, 20, 22, 36, 68, 71, 94, 95, 102, 113). The O_2_ microsensor falls into the amperometric category, which measures the current caused by the electrochemical reaction (an oxidation–reduction reaction) of O_2_ at the tip of the microsensor. In recent work on the measurement of O_2_ in sediments and biofilms, a miniaturized Clark-type O_2_ sensor with a guard cathode has been preferred to the cathode-type O_2_ microsensor (82, 95, 96, 105, 106, 107, 108, 110, 135).

### Construction and measuring principle

**Fig. 1:**
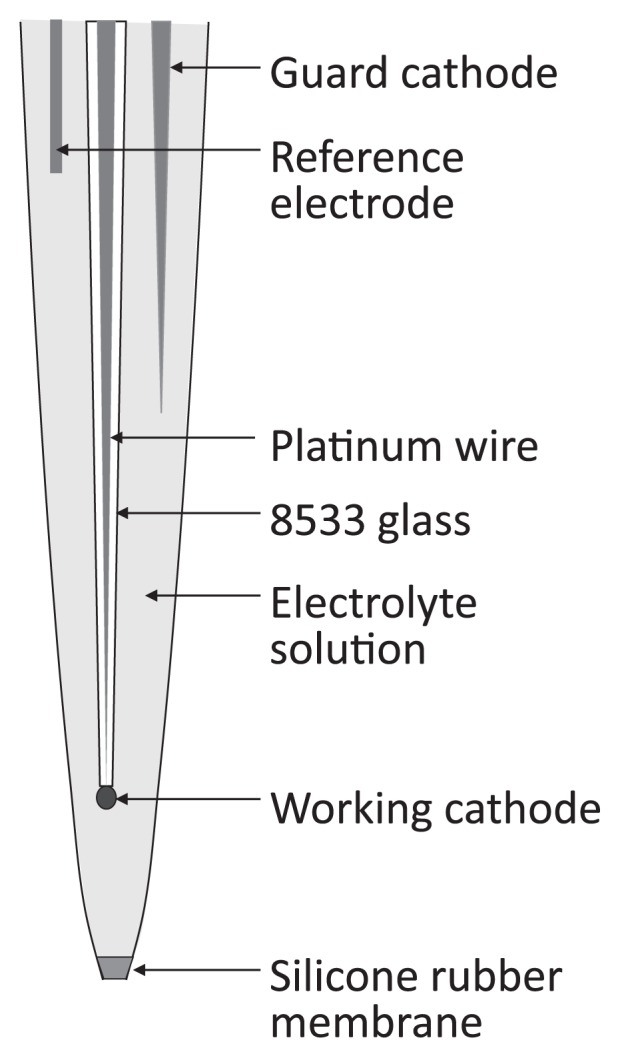
Schematic drawing of a Clark-type O_2_ microsensor.

**Fig. 2:**
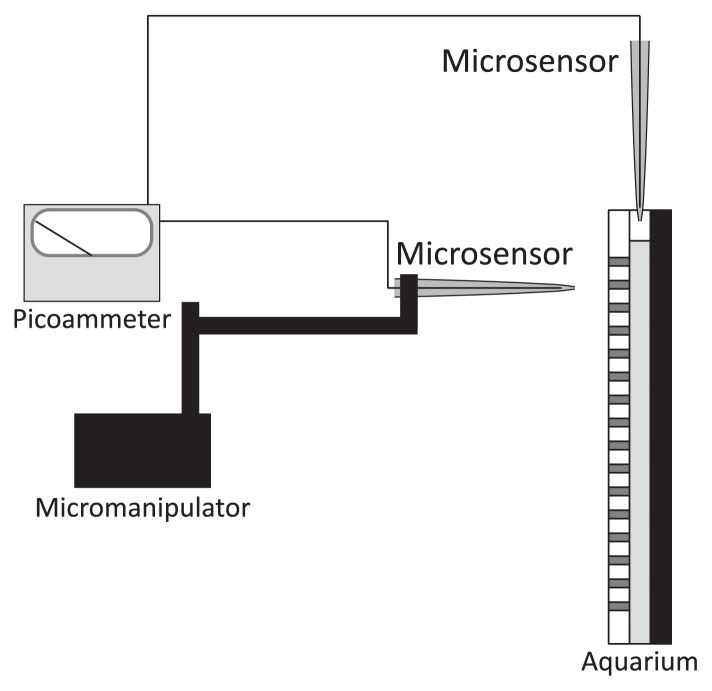
Experimental apparatus for monitoring O_2_ concentrations in sediment and an infaunal burrow.

In the Clark-type O_2_ microsensor (Fig. 1), the tip is coated with an electrically insulating membrane of silicone rubber, which is extremely permeable to O_2_ (96). The tip of a working cathode, which is a platinum wire electroplated with gold, is fixed just behind the membrane, otherwise its response time becomes too long. The microsensor is filled with an electrolyte solution of 1 M KCl, into which an internal guard cathode and a reference electrode, which are silver wires electrochemically coated with AgCl (Ag/AgCl wire), are immersed (95). If air bubbles remain in the microsensor, they must be removed so that vacuum conditions are in place. The working cathode, the reference electrode and the guard cathode are connected to a very sensitive ammeter (Fig. 2), termed a picoammeter, with a range down to 1 pA (Unisense A/S, Aarhus, Denmark) (http://www.unisense.com/). The electrochemical reaction is driven by the potential difference between the working cathode and the reference electrode. For O_2_ measurement, the working cathode is polarized by the battery to about – 0.8 V against the internal reference electrode. It should be noted that the polarization potential is specific to each type of amperometric microsensor (*e.g.*, O_2_, H_2_S, H_2_, N_2_O and NO) (27, 49, 95, 97, 113). Driven by external O_2_ partial pressure, O_2_ from the sample solution penetrates the membrane and is then reduced at the working cathode tip and thus the presence of O_2_ is registered. The current originating from the reduction of O_2_ at the working cathode tip should be proportional to O_2_ partial pressure in the surrounding solution. The picoammeter converts the reduction current to a signal.

Because of the small size of the cathode tip, diffusion is rapid relative to the convective transport of O_2_ to the cathode tip. Because of the small size and short diffusion path, the 90% response time can be less than 0.5 s (95). The electrolyte solution serves as electrical shielding for the working cathode to minimize electrical noise, allowing the O_2_ microsensor to be mounted on benthic landers for *in situ* measurements (37). The internal guard cathode is also polarized and removes all O_2_ diffusing toward the working cathode from the internal electrolyte reservoir, thus minimizing zero-current and polarization time. The signal from a typical O_2_ microsensor is much more stable than that from a cathode-type O_2_ microsensor, and current drift is 1% per hour (13). As the O_2_ microsensor responds linearly to changes in O_2_ concentrations, two-point calibration is sufficient (*e.g.*, zero mg L^−1^ O_2_ and full air saturation).

## Oxygen planar optodes

Although O_2_ concentration dynamics in burrows have been successfully monitored with O_2_ microsensors (10, 105, 121, 137), the monitoring of spatial and temporal O_2_ dynamics in the bioturbated sediment poses a further challenge. Problems arise in this case because of the patchiness of the macrobenthos and microorganisms, and their activities. Because the O_2_ microsensor measures O_2_ concentration at a single point, one-dimensional O_2_-concentration profiles at several distinct positions are obtained by stepwise insertion in the sediment. Therefore, from these one-dimensional results it is difficult to extrapolate two- or three-dimensional O_2_ distributions in complex bioturbated sediment on larger scales (centimeter to meter scales). Simultaneous measurement of O_2_ concentrations at several points requires a series of O_2_ microsensors with associated recording devices, which is expensive and impractical in most cases. Thus, due to limited horizontal resolution, attributed to the one-dimensional nature of O_2_ microsensors, monitoring two-dimensional O_2_ distribution in bioturbated sediments using O_2_ microsensors is a very difficult and time-consuming, if not impossible, task. Furthermore, measuring several O_2_ profiles at several points in the sediment in order to simultaneously describe temporal O_2_ dynamics is laborious, especially under non-steady-state conditions. Recently, however, the development of planar optodes has enabled us to visualize two-dimensional O_2_ distribution in the sediments.

### Indicators

The setup for planar-optode measurement has previously been described in detail (40, 46) and is thus only briefly presented here. The measuring principle behind the O_2_ planar optode is based on the dynamic quenching of a luminescent fluorophore by O_2_ (O_2_ indicator), where O_2_ decreases the fluorescence quantum yield of the O_2_ indicator (40, 46, 51). The most frequently employed O_2_-quenchable f1uorophores are ruthenium(II)-tris-4,7-diphenyl-1,10-phenathroline (28, 40, 46, 101, 116, 117, 134), platinum(II)-meso-tetra(pentafluorophenyl)porphyrin (32, 116), and platinum-octaethyl-porphyrine (46). Staal *et al.* (116) recently developed a new transparent optode for life-time-based microscopic imaging of O_2_, which is based on the use of iridium(III) acetylacetonato-bis(3-[benzothiazol-2-yl]-7-[diethylamino]-coumarin) as the O_2_ indicator. Compared to O_2_ optodes based on the ruthenium(II) polypyridyl complex or the platinum(II) porphyrin, the new planar optode has the advantages of being brighter, having a more homogeneous and smaller pixel-to-pixel variation over the sensor area, and having lower temperature dependency. These characteristics allow for much shorter exposure times and thus lead to very short response times, less noisy O_2_ image analysis and simplification of the calibration procedure. A short response time is critical in O_2_ optodes, for example, when the O_2_ concentration in a sample changes quickly under non-steady-state conditions.

### Construction and measuring principle

**Fig. 3:**
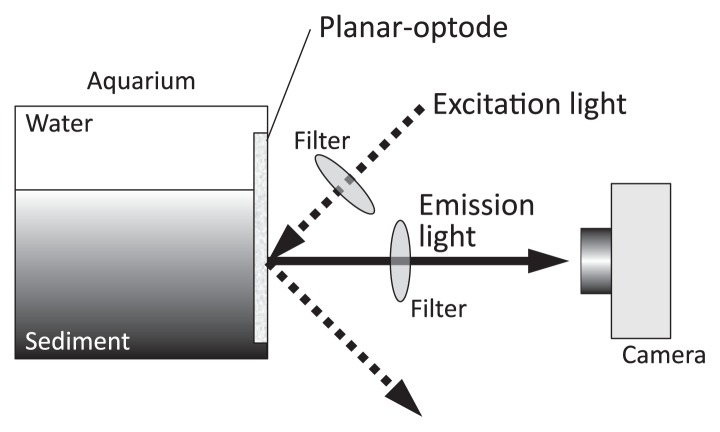
Schematic drawing of the experimental setup for a planar-optode measurement.

The O_2_ indicator is immobilized on a support foil, a microscope slide, a transparent polyethylene terephthalate carrier foil with plasticized PVC, or an organically modified sol-gel or polystyrene (28, 32, 40, 46, 101, 116, 134). The typical thickness of the O_2_ indicator layer in the planar optodes is less than 20 μm. The planar optode is placed on the inside of an aquarium wall (Fig. 3). Excitation light with specific wavelengths for each O_2_ indicator is supplied from the outside. The image of the O_2_-dependent fluorescent signal emitted by the planar optode is recorded by a digital charge-coupled device (CCD) camera, and thus yields a description of two-dimensional O_2_ distribution. Therefore, the spatial resolution of the O_2_ planar optode is dependent on the optical performance of the setup, properties of the indicators, and blurring of the signal due to oxygen diffusion in the O_2_ indicator layer (32). Spatial resolution can easily be changed by modifying the optical configuration in front of the CCD camera; for example, images covering an area of 26×25 mm and 70×50 mm correspond to spatial resolutions of 50 μm per pixel (54) and 105 μm per pixel, respectively (142).

In the presence of O_2_, the fluorescence intensity of the O_2_ indicator decreases predictably due to the quenching process (40). In contrast to the O_2_ microsensor described above, the calibration curve for the O_2_ planar optode, based on dynamic quenching of the luminescence of the O_2_ indicator, is nonlinear. Instead, the signal of the O_2_ planar optode can be described by the Stern-Volmer equation,

$$\frac{\text{I}}{I_{0}}=\frac{1}{1+K_{SV}C}$$

where *I_0_* and *I* are fluorescence intensities in the absence and presence of O_2_, respectively; *K_SV_* is the Stern-Volmer constant; and *C* is the O_2_ concentration (81). This simple Stern-Volmer equation is, however, only strictly valid for ideal systems, such as diluted solutions of fluorophore in a liquid solvent. Thus, slightly modified versions of the Stern-Vollmer equation are sometimes preferred because they more adequately describe the response of the optodes (32, 39, 46, 134).

In addition, the fluorescence lifetime (*i.e.*, decay time) of the O_2_ indicator can be used for O_2_ measurements (46, 131). In this case, the measuring principle relies on dynamic quenching of the indicator luminescence in response to O_2_. The decay time is a direct function of the phase of the luminescent light, which can be used directly for O_2_ detection. Luminescence-lifetime imaging has advantages over intensity-based imaging and allows enhancement of the contrast and background suppression of unwanted luminescence contributions in the image (46). Moreover, lifetime imaging does not depend on intensity variations due to photobleaching and variable indicator concentrations, and calibration-free sensing applications are also possible. The basic working principles of lifetime-based optodes can be found in previous reports (40, 46, 67, 131). In addition to the modifications described above, many different O_2_ indicators and imaging setups have been developed to meet the requirements of various specific experimental purposes (46, 54, 81, 92, 115).

## An example of fine-scale oxygen measurements in macrofaunal burrows with an O_2_ microsensor

### Introduction

In this section, an example of the application of O_2_ microsensors to measure O_2_ concentrations in burrows constructed by polychaete (*Tylorrhynchus heterochaetus*) is presented. Many studies have reported direct O_2_-concentration measurements in burrows without sampling (see next section) and have shown evidence of enhanced mass transport through the burrows. However, measurements with O_2_ microsensors were limited to depths of just a few centimeters from the sediment surface due to poor accessibility of the O_2_ microsensor, and the exact position of the burrow was unknown. Because the macrobenthos commonly live in deeper parts of the sediment (*e.g.*, >100 mm below the sediment surface) and play an important role in mass transport into the sediment, measurements of solute concentrations in deeper parts of the sediment are essential to investigate the effect of the macrobenthos on mass transport. Therefore, we studied O_2_ concentrations in burrows with O_2_ microsensors to provide direct evidence of mass transport into deeper parts of the sediment through burrows.

### Materials and methods

Sediment from the Niida River estuary in Hachinohe City, Japan, was selected, in which a large number of burrows have been constructed by a macrobenthos, *Tylorrhynchus heterochaetus*, which inhabits the intertidal zone of Japanese estuaries. To directly measure O_2_ concentration profiles in the burrows and bulk sediment, we constructed a continuous-flow aquarium with agar slits in one side (105). River water and sediment samples were collected in the intertidal area of the Niida River, which is located approximately 1.5 km from the river mouth (77, 78, 105). Grab samples of sediments were collected in November 2002. The sediment samples were passed through 1-mm mesh to remove pebbles, large detritus particles and indigenous macrobenthos.

**Fig. 4:**
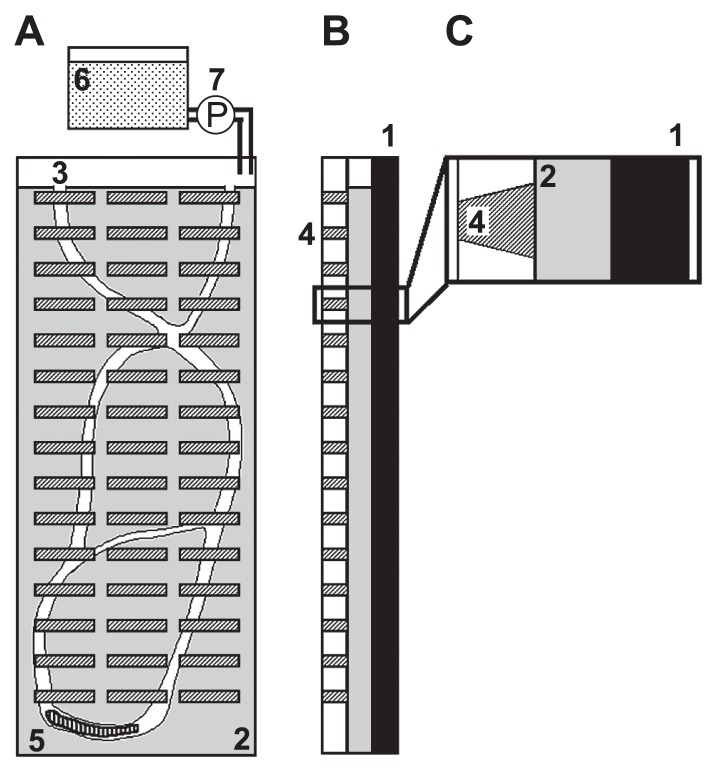
Schematic drawing of an aquarium: 1, sideboard; 2, sediment; 3, infaunal burrow; 4, agar plate; 5, an infauna; 6, tank filled with river water; 7, pump. (A) Front view. (B) Side view. (C) Close-up view of the side view enclosed by the box in panel B.

The concentration profiles of O_2_ and oxidation-reduction potential (ORP) were measured in the laboratory using microsensors, as described by Satoh *et al.* (105). The microsensors for ORP, which were made from a platinum wire coated with a glass micropipette, were constructed and calibrated as described by Jang *et al.* (48). All ORP data reported in this paper were relative to the Ag/AgCl reference sensor. To directly determine O_2_ concentrations around and inside the burrows, we constructed an aquarium from acrylic plates (105). Forty-five slits (5×50 mm) filled with 3% agar plate were made in one side of the aquarium (Fig. 4), allowing us to determine the burrow structure and microsensor position in the burrow. The aquarium was filled with the sediment collected at the study site. When a macrobenthos (*T. heterochaetus*) was placed on the sediment surface in the aquarium, it immediately began to dig a burrow in the sediment. River water was continuously fed through the aquarium at a flow rate of 2 mL min^−1^. The aquarium was maintained at 20°C and in dark conditions. After 3 d, the macrobenthos had created visible burrows down to 400 mm.

To measure O_2_ concentrations in the burrow or bulk sediment in deeper parts of the sediment, the O_2_ microsensor was inserted into the burrow through the agar plate to the center of the aquarium (*i.e.*, 10 mm from the agar surface). An O_2_ microsensor was mounted on a motor-driven micro-manipulator (model ACV-104-HP; Chuo Precision Industrial Co., Ltd., Tokyo, Japan) (Fig. 2). O_2_ concentration profiles in the biofilms were obtained by using the micromanipulator at intervals of 50 to 500 mm from the agar surface (or bulk liquid) into the sediment. The position of the microsensor tip was determined with a dissecting microscope (model Stemi 2000; Carl Zeiss). In order to determine O_2_ concentrations inside the burrow, on the burrow wall (*i.e.*, the burrow–sediment interface) and in the bulk sediment, the O_2_ microsensor was inserted into four different points in the sediment, positioned laterally at a depth of 80 mm from the sediment surface (see Fig. 2). In addition, the O_2_ microsensor was inserted from the top of the aquarium to measure a two-dimensional contour plot of O_2_ concentrations at the sediment surface (Fig. 2). Eleven vertical O_2_-concentration profiles were measured at 0.5 mm to 3 mm intervals along a transection of the sediment surface. A contour plot was constructed from the profiles. Microprofiles for O_2_ and ORP were determined only once at different positions in the sediment.

The profiles of ORP levels were also measured at a cross section of the sediment in a flow cell filled with a synthetic medium (105). To ensure that steady-state profiles were obtained, the sediment was incubated in the medium at 20°C for more than 30 min before taking the measurements. The details of the measurements are described elsewhere (105).

### Results

**Fig. 5:**
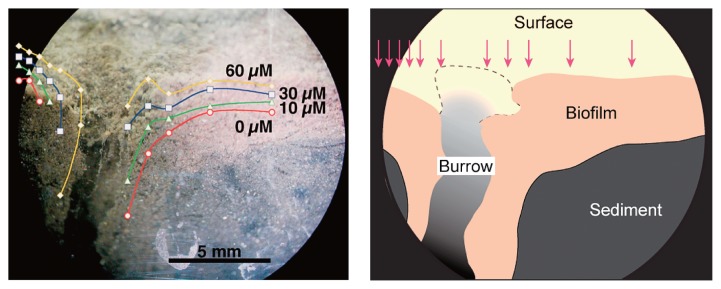
A representative two-dimensional contour plot of O_2_ concentration in the sediment (left) and drawing of a cross section of the sediment indicated in the left panel. A microsensor was inserted at 11 points (arrows in the right panel).

Many burrow openings (approximately 2000 ind. m^−2^), with diameters of approximately 5 mm, were found on the sediment surface at the study site during low tide. The visible burrows extended down to a depth of at least 400 mm. The burrow walls were covered with thin oxidized light-brown layers (Fig. 5) (105). The color of the burrow wall was similar to that of the sediment surface. Further information on the sediment characteristics at the study site and the physical and chemical parameters in the river water can be found elsewhere (77, 78, 105).

A representative two-dimensional contour plot of O_2_ concentrations at the sediment surface, constructed from 11 vertical O_2_ concentration profiles, is shown in Fig. 5. In this case, the O_2_ concentration in the overlying water was ca. 80 μM. The contour plot of O_2_ around the burrow opening is parallel to the burrow structure, demonstrating that O_2_ was transported into the sediment through the burrow. Fluffy brown biofilm developed on the surface and burrow wall. Biofilm thickness was ca. 5 mm. During the microsensor measurements, we occasionally found that suspended particles flowed in the burrow and the fluffy biofilm on the burrow wall oscillated, which proved that water did actually flow through the burrow.

**Fig. 6:**
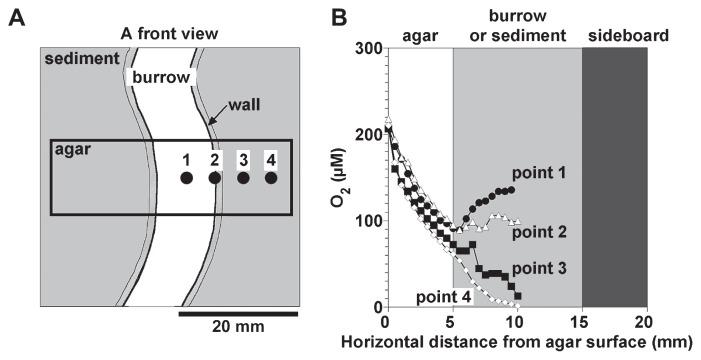
Horizontal O_2_ concentration profiles in the burrow and in the surrounding bulk sediment at a depth of 80 mm from the sediment surface.

The O_2_ microsensor was inserted horizontally through the agar plate of the aquarium into 4 different points in the sediment at a depth of 80 mm from the sediment surface (Fig. 6A). Fig. 6B shows O_2_-concentration profiles in the burrow and in the surrounding bulk sediment. In this case, the O_2_ concentration in the overlying water was ca. 190 μM. The O_2_ concentration of ca. 210 μM at the agar surface decreased to 60–90 μM in the agar plate. When the O_2_ microsensor was inserted at the center of the burrow (point 1 indicated in Fig. 6A), the O_2_ concentration increased toward the center of the burrow (at a point 10 mm from the agar surface in Fig. 6B). In contrast, the O_2_ concentration remained almost unchanged at the burrow–sediment interface (point 2). O_2_ concentrations in the surrounding bulk sediment decreased further, down to less than 10 μM at a point 10 mm from the agar surface (points 3 and 4). These results clearly show the horizontal O_2_-concentration profile from the center of the burrow to the surrounding anoxic bulk sediment.

**Fig. 7:**
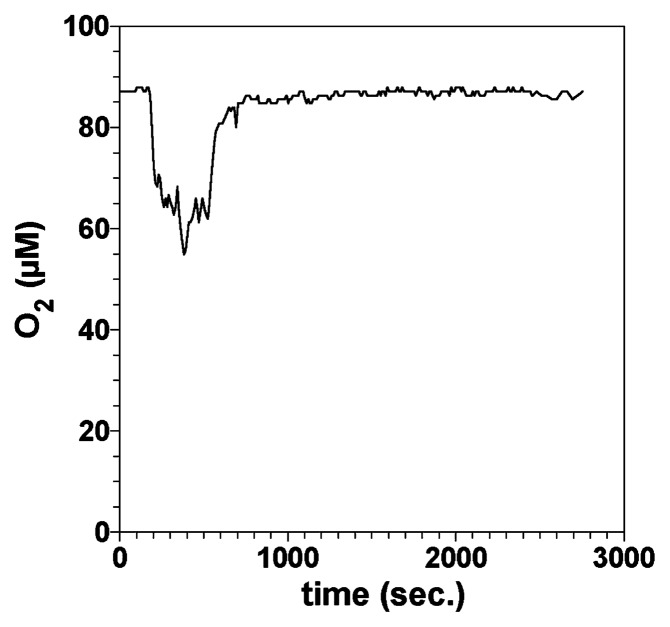
Temporal changes in O_2_ concentration in the burrow.

Temporal changes in the O_2_ concentration in the burrow were monitored by inserting and fixing an O_2_ microsensor at the center of the burrow (*i.e.*, 10 mm from agar surface) at a depth of 170 mm from the sediment surface, and continuously recording O_2_ concentrations (Fig. 7). In this case, the O_2_ concentration in the overlying water was ca. 190 μM. The O_2_ concentration was ca. 90 μM when the O_2_ microsensor was inserted (0 s). The O_2_ concentration started to decrease at 170 s, reached the minimum value (ca. 55 μM) at 380 s, and then finally returned almost to the initial level (ca. 85 μM) at 750 s. Interestingly, we found that the macrobenthos wriggled during the period when the O_2_ concentration decreased. Movement of the macrobenthos and changes in the O_2_ concentration in the burrow were not observed for at least 2,000 s after the initial 750 s. The burrows of the macrobenthos extended into anoxic sediment; thus, they must pump overlying oxic water through their burrows to meet their respiratory needs. Burrow irrigation can also be required for feeding, metabolite removal, and avoidance of the toxic effects of sulfide in anoxic pore waters (34). Bioirrigation introduces O_2_ and nutrients into an otherwise anoxic sediment, which enhances solute exchange between the water column and pore waters, thereby influencing the biogeochemical cycling of nutrients.

Many studies have aimed to measure solute concentrations in burrows with microsensors (11, 38, 72, 73, 138); however, all of the data were limited to the upper parts of the sediments (a few cm deep). In this study, we constructed and used an aquarium with agar slits in a side panel to overcome this limitation. To determine the O_2_ concentration profile along the burrow structure, the O_2_ microsensor was inserted through the agar slits of the aquarium into the center of the burrow to different sediment depths (105). In this case, the O_2_ concentration in the overlying water was ca. 190 μM. The O_2_ concentration in the burrow decreased from this value to 120 μM at a depth of 80 mm due to respiration of the macrobenthos and microorganisms, but below this depth, the decrease in O_2_ concentration was moderate. Interestingly, O_2_ still existed in the burrow even at a depth of 350 mm (105). This was attributed to the irrigation activity of the macrobenthos to exchange water with low concentrations of O_2_ and metabolites with the overlying fresh water (Fig. 7) (58). In contrast, the O_2_ penetration depth was only a few millimeters into the sediment surface around the burrow opening (Fig. 5). Using the newly developed aquarium, we could directly determine, for the first time, the O_2_ concentration profile in the burrow down to a depth of 350 mm from the sediment surface. This result provided direct evidence of the contribution of the macrobenthos to O_2_ transport, through the burrow, to deeper sediment.

Our aquarium had the advantages of being able to observe the exact positions of the burrows in the sediment and to directly measure the O_2_ concentration profiles in and around the burrows in deeper parts of the sediment. The main disadvantage was, however, that O_2_ diffused into the burrow and sediment through the agar plate, as indicated by the O_2_ concentration gradients in the agar plate (Fig. 6B). The O_2_ transport rates through the agar plate corresponded to ca. 5% of the total O_2_ consumption rates in the burrow wall (105). Thus, O_2_ concentrations in the burrow were slightly overestimated in this study; however, this does not negate the observed trends in the results presented here.

**Fig. 8:**
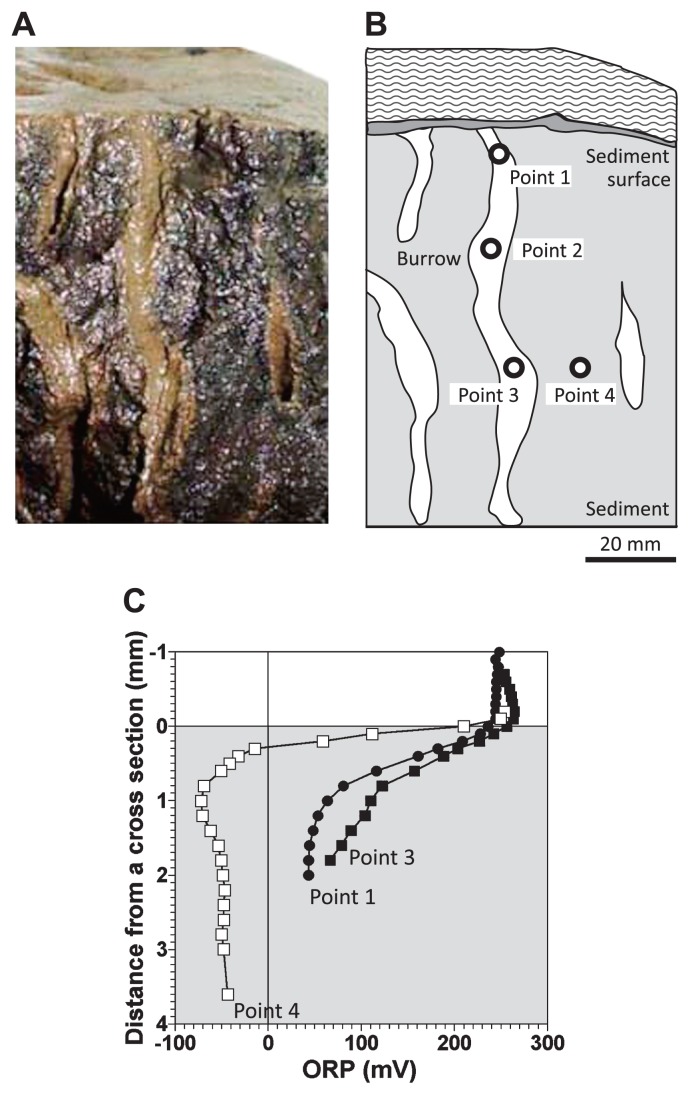
ORP profiles at 3 points (Point 1 [●], Point 3 [■], and Point 4 [□]) in a cross section of the sediment. (A) Photograph of the cross-section of the sediment. (B) Drawing of the cross-section of the sediment indicated in panel A. Points 1 to 4 indicate the points where the microelectrodes were inserted. (C) ORP profiles at 3 points. Zero on the horizontal axis corresponds to the surface of the cross-section.

Moreover, steady-state ORP profiles on burrow walls were measured in a cross-section of the sediment, which was collected without disturbing the physical structure of the cross-section of the sediment at the same sampling site (105). ORP microsensors were inserted into 3 points of the cross section; the burrow wall at depths of 5 mm (point 1) and 50 mm (point 3) and the bulk sediment at a depth of 50 mm (point 4) from the sediment surface (Fig. 8B). The ORP level declined sharply in the upper 0.5 mm of the bulk sediment (point 4) in comparison to at the burrow wall (points 1 and 3) (Fig. 8C). ORP levels at a depth of ca. 2 mm from the cross section were ca. +50 mV at the burrow wall (points 1 and 3), whereas ca. −30 mV in the bulk sediment (point 4), indicating that the burrow-wall sediment was more oxygenated than the bulk sediment. These results clearly indicate that the layered structure of oxygenated and reduced zones, found at the sediment surface, was created at the burrow wall.

The higher levels of O_2_ and ORP in the burrow walls altered the abundance, diversity and activity of ammonia-oxidizing bacteria (AOB) and nitrite-oxidizing bacteria (NOB) in the burrow walls rather than in the bulk sediment (105). Real-time quantitative PCR (Q-PCR) assay demonstrated that AOB and *Nitrospira*-like NOB-specific 16S rRNA gene copy numbers in the burrow walls were comparable with those in the sediment surfaces and higher than those in the bulk sediment at the same depth. The 16S rRNA gene-cloning analysis revealed that betaproteobacterial AOB communities in the sediment surface and burrow walls were dominated by *Nitrosomonas* sp. strain Nm143-like sequences. The second most frequently detected clones recovered from the sediment surface were affiliated with the *Nitrosomonas marina* lineage, whereas they were affiliated with the *Nitrosospira briensis* lineage in the burrow walls. Microelectrode measurements showed higher NH_4_^+^ consumption activity at the burrow wall than in the surrounding sediment. These results clearly demonstrated that the infaunal burrows stimulated O_2_ and mass transport into the sediment in which otherwise reducing conditions prevailed, resulting in the development of high NH_4_^+^ consumption capacity. Consequently, the infaunal burrow became an important site for NH_4_^+^ consumption in the intertidal sediment.

## Spatial and temporal dynamics of oxygen concentrations in macrofaunal burrows: a review of the recent literature

In previous studies, spatial and temporal O_2_ dynamics in macrofaunal burrows in sediments were measured with O_2_ microsensors and O_2_ planar optodes at high spatial and temporal resolution. Table 1 summarizes these papers, listing the macrobenthos species that created the burrows and the range of O_2_ concentrations in the burrows. To date, studies to investigate O_2_ concentrations in the burrows have focused mainly on the burrows created by polychaetes and insect larvae, which inhabit sediments in estuarine, lake or river waters.

O_2_ was introduced into burrows in deeper sediment layers by the activities of the macrobenthos (*e.g.*, bioirrigation). Microsensor measurements demonstrated that O_2_ was present in the burrows, at least at the points where the O_2_ microsensor was inserted (at depths of 3.5 to 50 mm). O_2_ concentrations in the burrows differed depending on the burrow inhabitant. Furthermore, O_2_ planar optodes allowed O_2_-concentration measurements in deeper parts of the sediments (to depths of 500 mm) than when using O_2_ microsensors. It should be noted that our aquarium also allowed for O_2_ measurements in burrows in deeper parts of the sediment (to depths of 350 mm) (105).

O_2_ concentrations in the burrows fluctuated over time and ranged from 0% to 100% of air saturation in response to bioirrigation activity of the macrobenthos (Table 1). The burrows were intermittently irrigated in a sequence of pumping events by the macrobenthos, followed by a period of rest, which could be interpreted from the temporal variation in the O_2_ concentration (91). In our study, the time interval between the initial O_2_ decrease, due to the discharge of water with lower O_2_ concentration and with metabolites from the macrobenthos and microorganisms, and the moment the concentration returns to its peak after replacement with fresh oxygenated water, is considered to be the duration of the pumping event (170 s to 750 s in Fig. 7). The duration of the maximum O_2_ concentration is considered to be the rest period. Due to discontinuous irrigation, O_2_ concentrations in the burrows were highly variable over a timescale of minutes. The dynamic range of the O_2_ concentrations in the burrows and the cycle of pumping and resting events, differed depending on the macrobenthos species (138) and temperature (6), probably because the activities of the macrobenthos vary among species and with the environmental conditions in the burrows.

Macrobenthos are quantitatively important with respect to enhancing the area of the oxic–anoxic interface in sediments with their burrowing activity. Satoh *et al.* (105) studied the burrows created by *Tylorrhynchus heterochaetus* with ca. 5 mm diameter and roughly estimated that the area of the oxic–anoxic interface in the upper 350 mm of bioturbated sediment was approximately 9 times higher than the area of the sediment surface. Likewise, the presence of thalassinidean shrimp burrows increased the area of the oxic–anoxic interface by up to 9 times (43). A similar expansion of the area of the oxic–anoxic interface has also been demonstrated previously (Table 2). It is obvious that the increase in the area of the oxic–anoxic interface is dependent on the identity, density, mode of life and habits (feeding strategy, tolerance for low O_2_ concentration and tolerance towards toxic H_2_S and NH_4_^+^), and depth distribution of the macrobenthos. Teal *et al.* (130) calculated the global volume of bioturbated sediment to be 20,700 km^3^, based on a conservative estimate of the ocean area of 360 million km^2^, indicating a significant impact of the macrobenthos activities on chemical environments in sediments on a global scale.

Microsensors can detect O_2_ concentrations in the burrows directly and with minimum disturbance and a rapid response. As other types of microsensors (for H_2_S, H_2_, N_2_O, NO, pH, redox level, and flow-velocity measurement) are commercially available (Unisense A/S) and liquid ion-exchange (LIX)-based microsensors (NH_4_^+^, NO_2_^−^, NO_3_^−^ and pH) are easy to construct (23), microsensor measurements enable us to analyze nitrogen and sulfur cycles, as well as the O_2_ dynamics in macrofaunal burrows. The detailed quantitative analysis of O_2_ concentrations in the burrows with an O_2_ microsensor was limited to depths of 50 mm from the sediment surface, due to low accessibility of the microsensor and uncertainty of the exact position of the burrows. On the other hand, the O_2_ planar optode allowed O_2_-concentration measurements in deeper parts of the sediment than possible with the O_2_ microsensor (Table 1). In addition, the O_2_ planar optode allows for two-dimensional time-lapse measurements; however, the O_2_ planar optode is not always in direct contact with the burrows, resulting in the underestimation of O_2_ concentrations in the burrow. Likewise, the optode could not detect O_2_ in either the feeding pocket or in the advective zone (134). In addition, O_2_ measurements in sediments with the O_2_ planar optode potentially result in physical changes to the sediment structure due to insertion of the optode into the sediment (6). Photobleaching of the indicator can hinder reliable and long-term measurements. The O_2_ microsensor also infrequently suffers from drift. Consequently, the choice of appropriate tools is an important prerequisite to accurately measure O_2_ concentrations in the bioturbated sediment. The combined use of an O_2_ planar optode and O_2_ microsensor allowed for more accurate and reliable measurements of O_2_ concentrations in sediments (41, 90).

## Conclusions and future directions

During the last couples of decades, much effort has been devoted to investigating O_2_ concentrations in bioturbated sediments. Currently, O_2_ concentrations can be measured at high spatial and temporal resolution with O_2_ microsensors and O_2_ planar optodes. Extensive studies have provided considerable insight into O_2_ distributions and their spatial and temporal dynamics in macrofaunal burrows in sediments, as described above. Microsensor measurements clearly demonstrate that the macrofaunal burrow facilitates O_2_ transport into deeper sediment, in which otherwise reducing conditions prevail. O_2_ was detected in macrofaunal burrows at depths of 500 mm in the sediment, whereas the O_2_-penetration depth at the sediment surface was only a few millimeters. Thereby, the area of the oxic–anoxic interface in the sediments was enhanced by up to 9 times that of the area of the sediment surface as a result of the burrowing activity of the macrobenthos. Moreover, O_2_ distribution patterns were spatially and temporally dynamic in response to a sequence of pumping events by the macrobenthos followed by a period of rest.

However, O_2_ microsensors and O_2_ planar optodes have only been used to a very limited extent in studies concerning the distribution, transport and dynamics of O_2_ in macrofaunal burrows and sediments; therefore, up until now, little has been known about the biogeochemical cycles of carbon, nitrogen and sulfur in the bioturbated sediment. Future investigations should aim to map the concentrations of CO_2_, CH_4_, NH_4_^+^, NO_2_^−^, NO_3_^−^, NO, N_2_O, H_2_S, metals and the pH in the burrows. Planar optodes selective for CO_2_ (115, 147), NH_4_^+^ (128) and pH (114, 118, 146) are available; however, the types of indicators for planar optodes are limited. Hafuka *et al.* (44) reported a fluorescent molecule capable of recognizing heavy-metal ions, which suggests the possibility of developing corresponding planar optodes. In contrast, microsensors for H_2_S, H_2_, N_2_O, NO, pH, and redox potential are commercially available (Unisense A/S), and LIX-based microsensors (NH_4_^+^, NO_2_^−^, NO_3_^−^ and pH) are easy to construct (23, 36). *In situ* two-dimensional distribution of H_2_S has also been determined by diffusive gradients in the thin films (DGT)-computer-imaging densitometry (CID) technique (26). The combination of O_2_ microsensors and planar optodes with those for other dissolved gases and ions can enhance our understanding of the biogeochemical processes occurring in bioturbated sediments.

The biogeochemical processes in bioturbated sediments are not only affected by dissolved gases and ions, but also by many other factors, such as particle size and porosity in the sediment, transport properties (diffusion and advection), and the abundance, distribution and diversity of micro-organisms. Although a microsensor for flow-velocity measurements is commercially available (Unisense A/S) (100), and permeability can be estimated by nuclear magnetic resonance (NMR) (18), determination of physical properties in the sediment on the submillimeter scale is still difficult. Development of technology to determine the physical properties of burrows and sediments is urgently needed. On the other hand, the abundance and diversity of microbial communities has been frequently studied using molecular techniques, such as 16S rRNA gene clone libraries, FISH, DGGE and T-RFLP (25, 31, 35, 45, 50, 111, 129). Knowledge of these physical and/or microbial properties in burrows could be related directly or indirectly to the results obtained with O_2_ microsensors and O_2_ planar optodes.

Most of the studies discussed in this review were conducted in experimental mesocosms in laboratories. Only a few studies have performed *in situ* measurements of O_2_ concentrations in sediment, accomplished using autonomous benthic-lander systems that carry benthic chambers and profiling units (37, 41, 142). Although the *in situ* temperature and bottom-water O_2_ concentrations of the recovered sediment cores were maintained as closely as possible in the laboratory, the measurements introduced artifacts that affected O_2_ distribution, as well as microbial activities due to the disturbance of the sediment structure during core sampling and improper establishment of *in situ* conditions during the measurements. Hence, trustworthy results can only be obtained by *in situ* analysis.

## Figures and Tables

**Table 1:** Summary of O_2_ concentrations and their temporal fluctuations in burrows created by different macrobenthos species reported in previous studies. O_2_ concentrations were determined with an O_2_ microsensor or O_2_ planar optode. The macrobenthos species and the location of the study site are presented. Each source is listed by number in the literature cited

Species	Location	O_2_ concentration (μM or %)^a^	Temporal fluctuation (μM or %)^a^	Ref.
O_2_ microsensors				
*Tylorrhynchus heterochaetus* (polychaete)	Intertidal area, Japan		55–90 at 170 mm	This study
		70 at 350 mm		105
*Neotrypaea californiensis* (ghost shrimp)	Shallow lagoon, USA	45 at 3.5 mm	0–350 at 0 mm	10
*Hexagenia limbata* (mayfly)	Lake Saint Joseph, Canada	350 at 12 mm	0–350 at 0 mm	34
*Sialis velata* (alderfly)			0–200 at 4 mm	
*Chironomus riparius* larvae (diptera)	Freshwater sediments	<200 at 8 mm	0–200 at 6 mm	121
			0–100 at 8 mm	
			0–300 at 50 mm	
*Arenicola marina* (polychaete)	Near-coastal sandflat in fjord, Denmark	5–100% at 50 mm	—	134
*Campsurus notatus* (ephemeropteran)	Lake Batata, Brazil	200 at 44 mm	190–230 at 25 mm	64
*Ephoron virgo* larvae (mayfly)	River Rhine, Germany	190–230 at 25 mm	140–240 at 15 mm	120
			—	
Unidentified	Aarhus Bay, Denmark	50 at 9 mm	270–360 at 25–30 mm	38
*Hexagenia limbata* (mayfly)	Lake Saint Joseph, Canada	170 at 26 mm	20–380 at 25–30 mm	138
*Sialis velata* (alderfly)		270 at 23 mm	—	
*Corophium volutator* (amphipod), *Nereis* sp. (polychaete), and chironomid larvae	Inner, low-salinity part of a small fjord estuary, Denmark	170 at 6 mm	—	11

O_2_ planar optodes				
*Chironomus plumosus* larvae (diptera)	Shallow lake, Denmark and Germany		180–30 at 4°C at 20–30 mm	6
			170–100 at 15°C at 20–30 mm	
			120–0 at 21°C at 20–30 mm	
Earthworms	Peat soil, Denmark	50% at 35–45 mm	—	136
*Arenicola marina* (polychaete)	Intertidal area, Germany	40% at 150 mm	0–40% at 150 mm	125
*Nereis diversicolor* (polychaete)	Carteau cove, France	75% at 100 mm	—	89
*Nereis virens* (polychaete)	Channel, France	75% at 100 mm	—	
*Chironomus plumosus* larvae (diptera)	A freshwater lake, Germany	170 at 15 mm	20–120 at 12 mm	91
*Hediste diversicolor* (polychaete)	Harbour, Denmark	50% at 10 mm	0–90%	55
*Hediste diversicolor* (polychaete)	Shallow water subtidal site, Denmark	30% at 30 mm	—	142

a The concentrations are indicated as μM or percentage of air saturation.

**Table 2:** Summary of the ratios of the oxic sediment area and volume associated with burrows relative to the area and volume of oxic surface sediment, respectively, reported in previous studies. The macrobenthos species and the location of the study site are presented. Each source is listed by number in the literature cited

Species	Location	Ratio of area (−)	Ratio of volume (−)	Ref.
*Tylorrhynchus heterochaetus* (polychaete)	Intertidal area, Japan	9.1		105
*Hediste diversicolor* (polychaete)	Shallow water subtidal site, Denmark		0.54–0.7	142
*Hexagenia limbata* (mayfly)	Lake Saint Joseph, Canada		<0.26	138
Nereid polychaetes		1.3–5		57
*Nereis diversicolor* (polychaete), polychaetes (especially *Pygospia elegans*) and oligochaetes	Sandy sediment in harbour and bay, Denmark	2.5–5		29
Thalassinidean shrimp		9		43
